# Extremely Large Magnetic-Field-Effects on the Impedance Response of TiO_2_ Quantum Dots

**DOI:** 10.1038/s41598-019-41792-z

**Published:** 2019-03-29

**Authors:** Dominique Mombrú, Mariano Romero, Ricardo Faccio, Milton A. Tumelero, Alvaro W. Mombrú

**Affiliations:** 1 Centro NanoMat/CryssMat & Física, DETEMA, Facultad de Química, Universidad de la República (UdelaR), Montevideo, C.P. 11800 Uruguay; 2 Instituto de Física, Universidade Federal do Rio Grande do Sul (UFRGS), Porto Alegre, C.P. 91501-970 Brazil

## Abstract

Here, we report large magnetoresistance and magnetocapacitance response of undoped TiO_2_ quantum dots weighting the contribution of both grain and grain boundaries by means of impedance spectroscopy. We also performed a complete characterization of the TiO_2_ quantum dots (~5 nm) prepared by sol-gel via water vapor diffusion method, using X-ray diffraction, small angle X-ray scattering, transmission electron microscopy and Raman spectroscopy. In addition, we showed a complete theoretical study on the electronic properties of TiO_2_ surface and subsurface oxygen and titanium vacancies to shed some light in their electronic and magnetic properties. Based in our study, we can conclude that the presence of defects, mainly at the grain boundary of these undoped TiO_2_ quantum dots, could be responsible for the large positive magnetoresistance (+1200%) and negative magnetocapacitance (−115%) responses at low applied magnetic fields (1.8 kOe) and room temperature.

## Introduction

There is recent interest in the study of diluted magnetic semiconducting oxides after the theoretical prediction of room ferromagnetism in Mn-doped ZnO reported by Dietl *et al*.^1^ and experimentally evidenced for Co-doped ZnO by Ueda *et al*.^2^. However, after the evidence of ferromagnetism found for undoped hafnium dioxide (HfO_2_) films reported by Venkatesan *et al*.^3^, a lot of research has been focused in this field. For instance, ferromagnetism in TiO_2-d_ films was explained by the presence of oxygen vacancies, which consequently leads to the formation Ti^2+^ and Ti^3+^ ions with unpaired 3d electrons^4^. Sudakar *et al*. also showed experimental evidence of room temperature ferromagnetism in vacuum annealed undoped rutile TiO_2_ films^5^. In this case, ferromagnetism was attributed to the formation of an amorphous thin layer in the nanocrystalline surface, in comparison with the oxygen annealed specimens^5^. The theoretical approach to study the origin of oxygen vacancy induced ferromagnetism in bulk anatase and rutile TiO_2_ came afterward^6^. In this report, Kim *et al*. showed that oxygen-vacancy titanium neighbor atoms have a weak magnetic moment (0.06 μ_B_) in the case of anatase and a higher magnetic moment (0.22 μ_B_) for rutile polymorph^6^. On the other hand, Wang *et al*. recently showed p-type semiconducting behavior and room temperature ferromagnetism for undoped titanium-defect anatase Ti_1–X_O_2_ nanocrystalline samples^7^. In the same work, theoretical calculations by means of GGA + *U* methodology was also obtained for bulk titanium-defect anatase with Ti_15_O_32_ formula, evidencing the presence of a new band transition above the valence band in the density of states, which explains the higher mobility of the experimentally observed holes^7^. In a recent study, Bansal *et al*. have reported a small negative magnetoresistance response at 9 kOe of ultrathin SnO_2_ films at low temperatures (T < 40 K)^8^. Another report with Mn-doped ZnO films revealed large negative D.C. magnetoresistance response at 130 kOe and low temperatures (T < 20 K) in comparison with the low positive magnetoresistance observed for undoped case^9^. Moreover, Na-doped ZnO films has shown a transition from positive to negative magnetoresistance response at 3 kOe and room temperature with increasing amounts of dopant^10^. More recently, Shen *et al*. reported a transition from negative to positive magnetoresistivity for nitrogen-doped In_2_O_3_ films at 10 kOe and room temperature^11^. However, up to now, there are still very few reports about the electrical transport of diluted ferromagnetic semiconductors in response to relatively small applied magnetic fields, pursuing an enhancement of larger magneto-electric effect closer to room temperature and have considerable impact in terms of their applications. The major goal of the present report is to study the contribution of both grain and grain boundaries to the total magneto-impedance response in TiO_2_ quantum dots (~5 nm). In addition, we show a complete theoretical study on the electronic properties of TiO_2_ (101) surface and subsurface oxygen and titanium vacancies to shed some light in their electronic and magnetic properties.

## Results and Discussion

**Figure 1:**
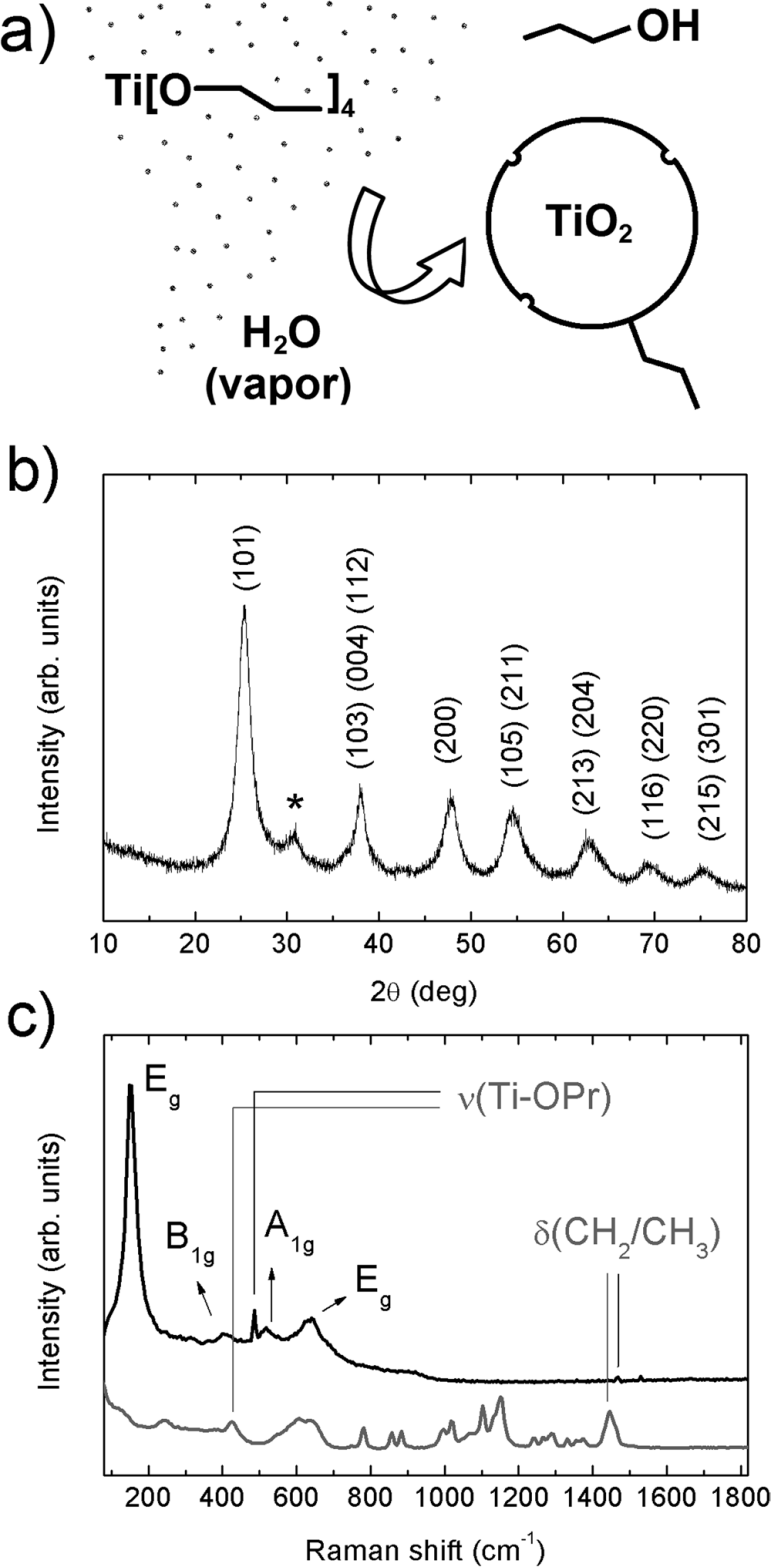
(**a**) Scheme of synthesis mechanism, (**b**) XRD pattern and (**c**) Raman spectra of TiO_2_-QDs samples. The *hkl* planes attributed to TiO_2_ anatase and TiO_2_ Brookite impurities (*) are shown in Fig. 1b. Raman modes attributed to residual *n*-propanoxide bonded to surface and an additional Raman spectra of the titanium n-propanoxide precursor are shown in Fig. 1c.

**Figure 2:**
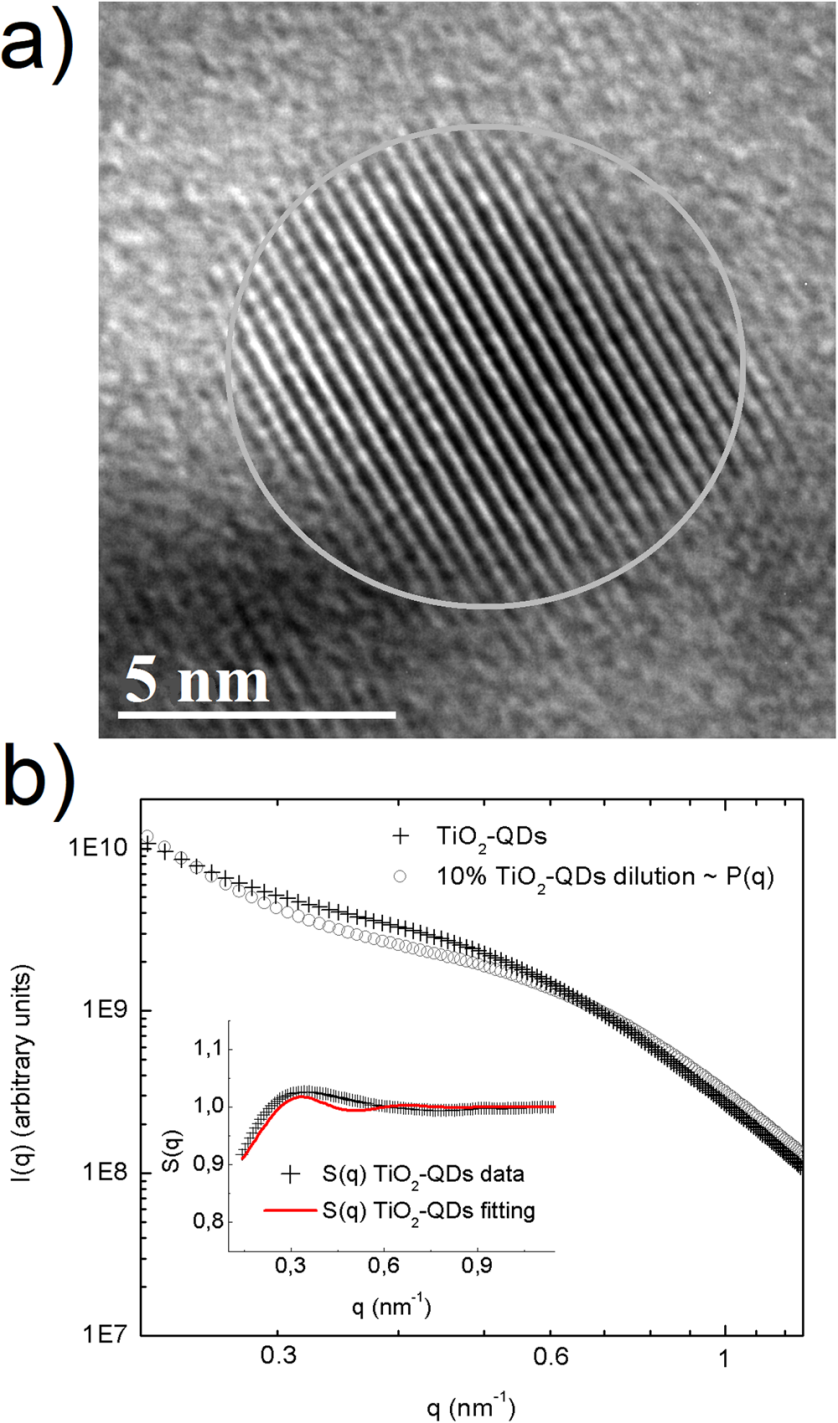
(**a**) HR-TEM image and (**b**) SAXS pattern for TiO_2_-QDs samples collected at T = 300 K. The total intensity I(q) for TiO_2_-QDs sample is displayed together with the corresponding experimental approximation to the form factor P(q) obtained using a 10% dilution of TiO_2_-QDs into a polymer host. The structure factor S(q) data for TiO_2_-QDs obtained as S(q) = I(q)/P(q) and S(q) fitting using the sticky hard sphere (SHS) approximation are shown in the inset.

**Figure 3:**
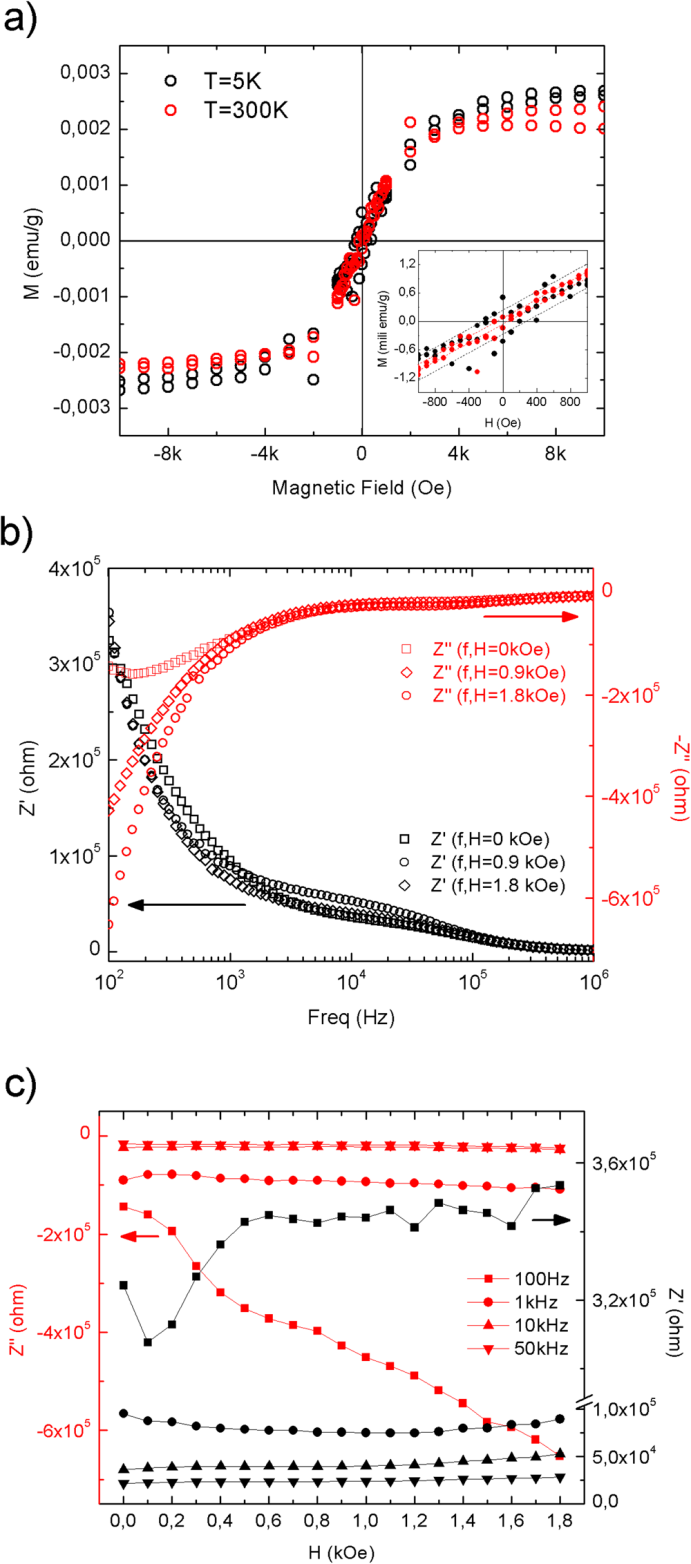
(**a**) Magnetization versus applied magnetic field for TiO_2_-QDs collected at T = 300 K and 5 K. Real (Z′) and imaginary (Z″) impedance (**b**) versus frequency at selected H = 0, 0.9 and 1.8 kOe magnetic fields and (**c**) versus applied magnetic field at 100 Hz, 1 kHz, 10 kHz and 50 kHz selected frequencies.

**Figure 4:**
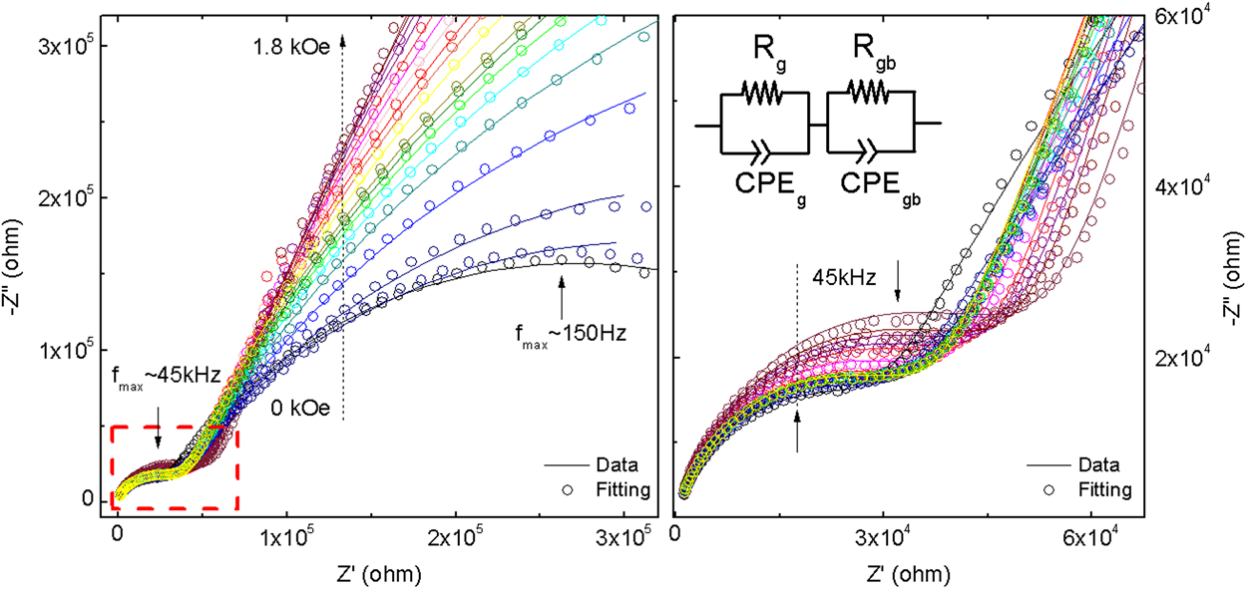
Nyquist plots data (circles) for impedance curves collected at different applied magnetic fields (H = 0–1800 Oe) for TiO_2_-QDs and corresponding fittings (lines) using the circuit model shown in the left panel. The dashed arrow indicates the increasing magnetic field from 0 to 1.8 kOe and the solid arrows indicate the approximated relaxation frequencies at the maximum of the arc for both processes at higher (~45 kHz) and lower (~100–150 Hz) frequencies. The right panel also shows a zoom of the low-impedance regime at high frequencies.

**Figure 5:**
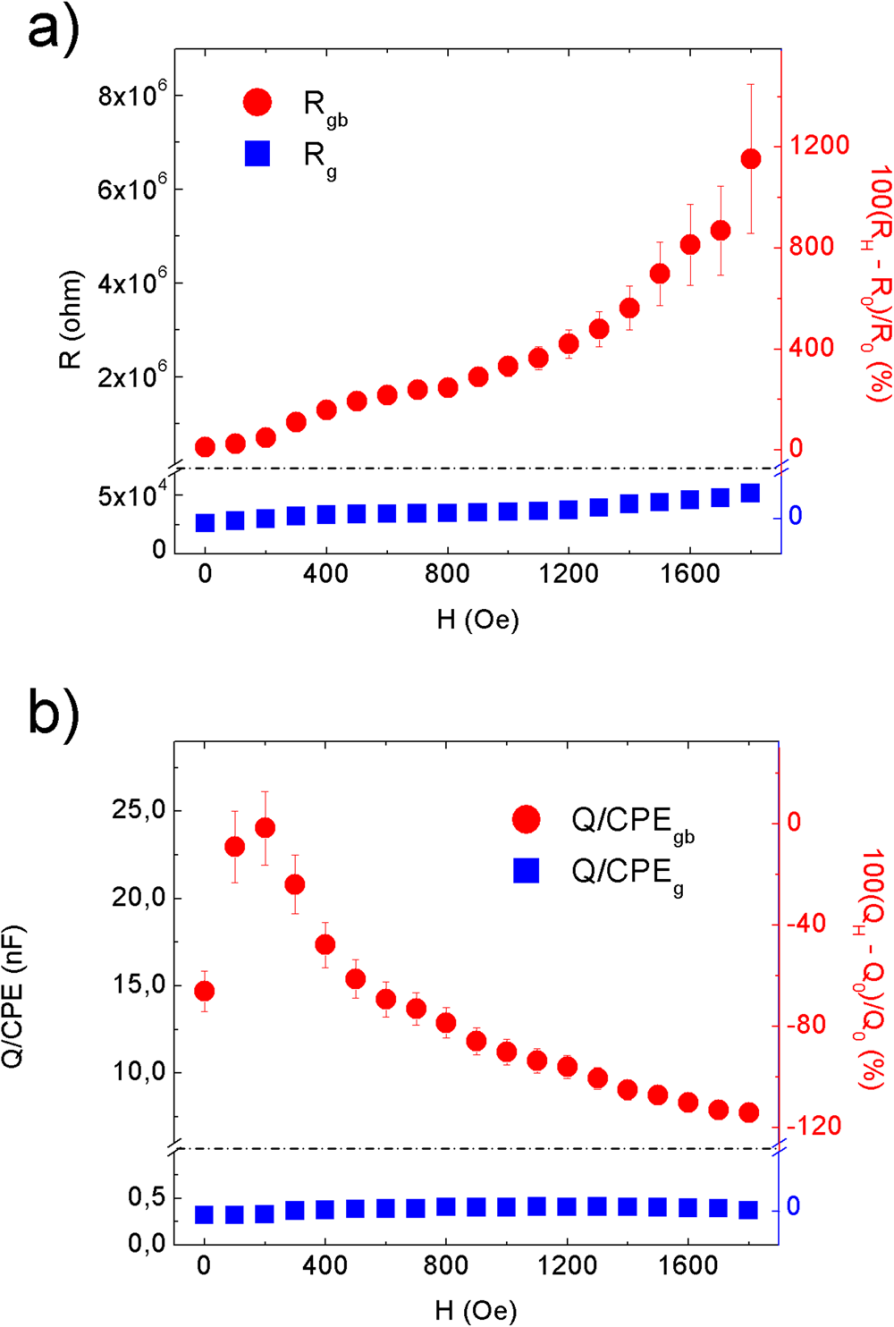
Resistance (R) and pseudo-capacitance (Q/CPE) fitted values for grain boundary and grain contributions to the TiO_2_-QDs as a function of the applied magnetic field. Magneto-resistance and magneto-capacitance response calculated as MR(%) = 100(R_H_ − R_0_)/R_0_ and MQ(%) = 100(Q_H_ − Q_0_)/Q_0_, respectively.

**Figure 6:**
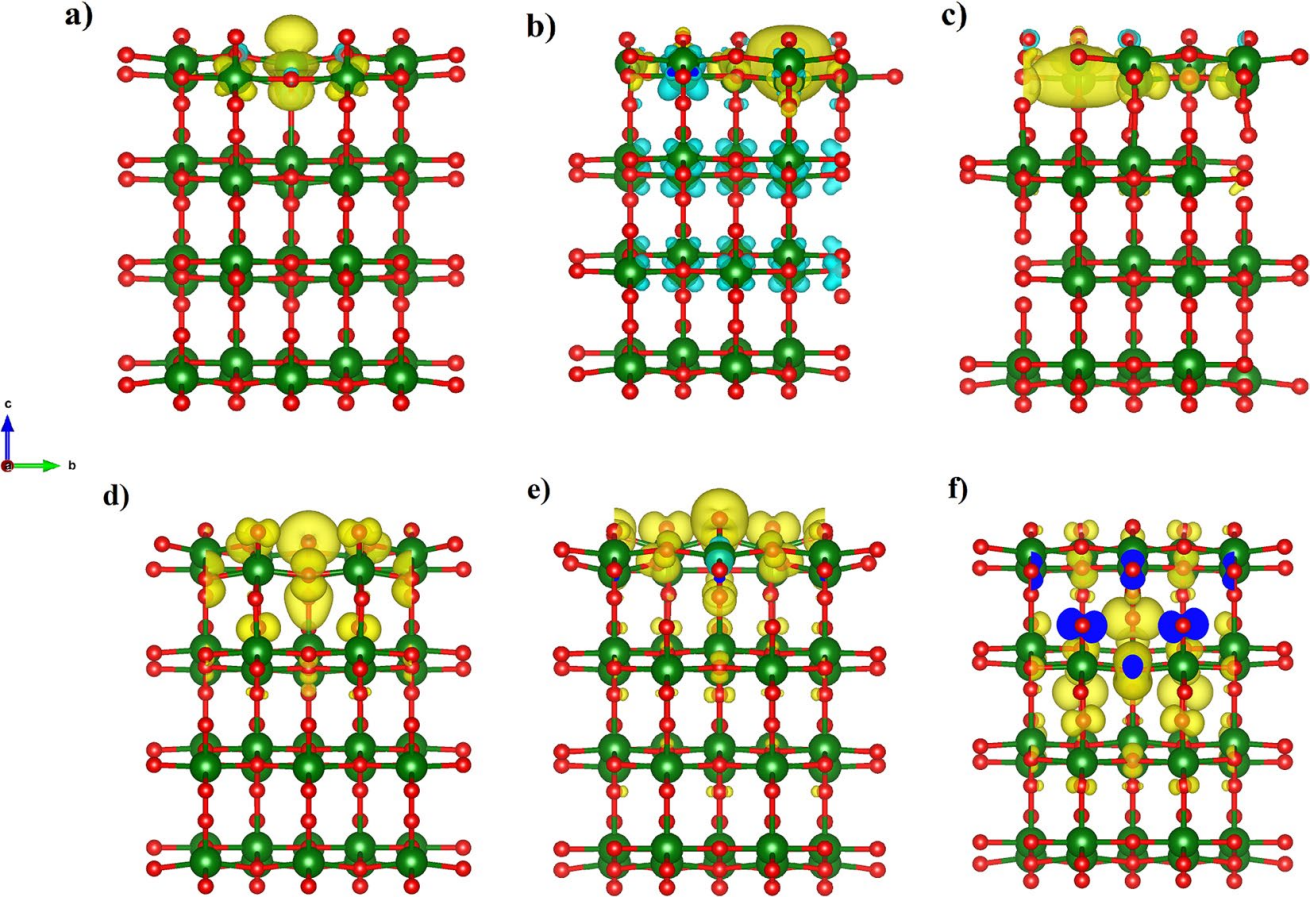
Spin-up and spin-down isosurface represented in yellow and cyan colors, respectively for (**a**) 1-O, (**b**) 2-O, (**c**) 3-O. Spin-up and spin-down isosurface at 50% of maximum value represented in yellow and cyan, respectively for (**d**) a-Ti, (**e**) b-Ti, (**f**) c-Ti.

**Figure 7:**
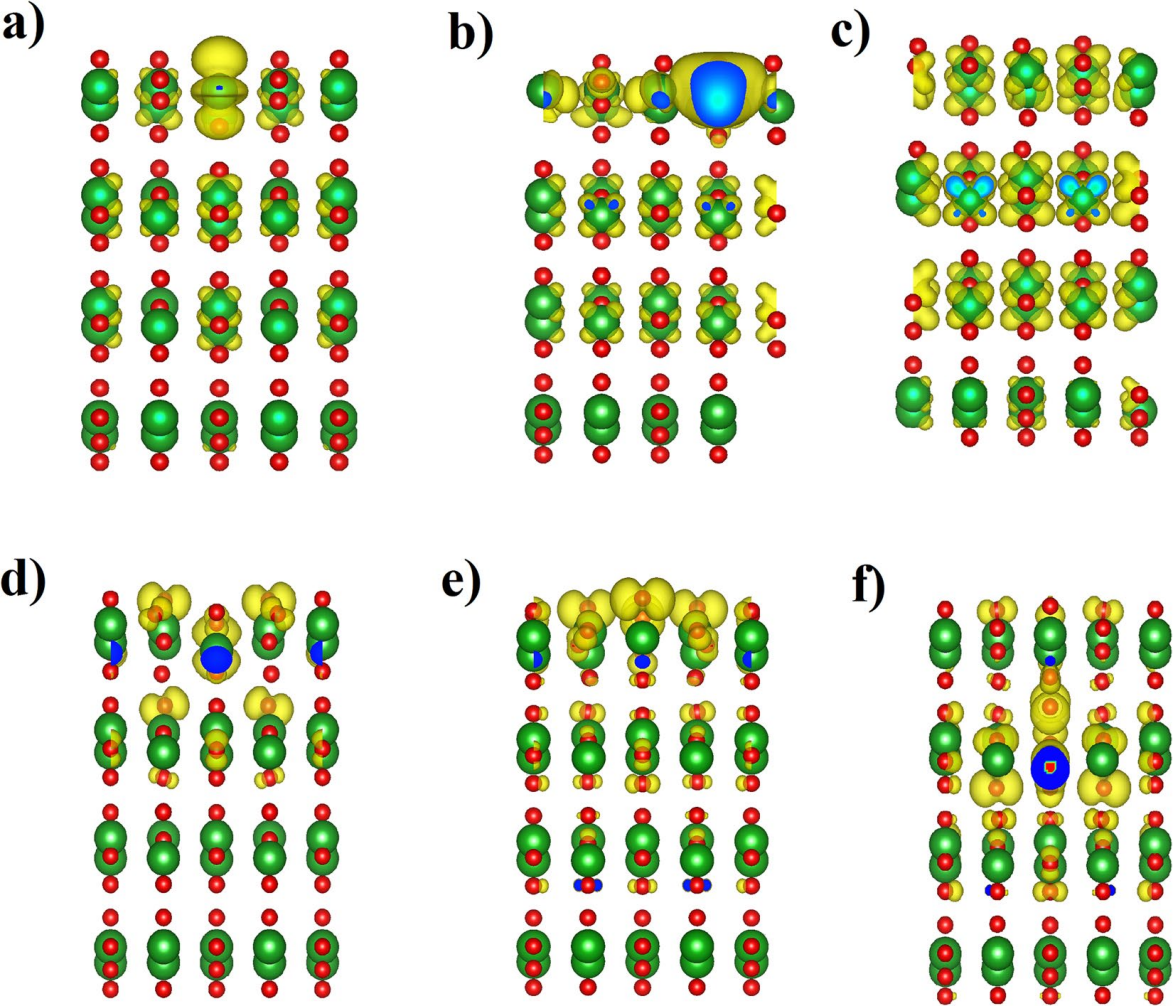
Charge density associated of the single electron wavefunctions, corresponding to energy ranges: −0.2 to 0.0 eV for (**a**) 1-O, (**b**) 2-O, (**c**) 2-O and 0.0 to 0.2 eV for (**d**) a-Ti, (**e**) b-Ti, (**f**) c-Ti.

**Figure 8:**
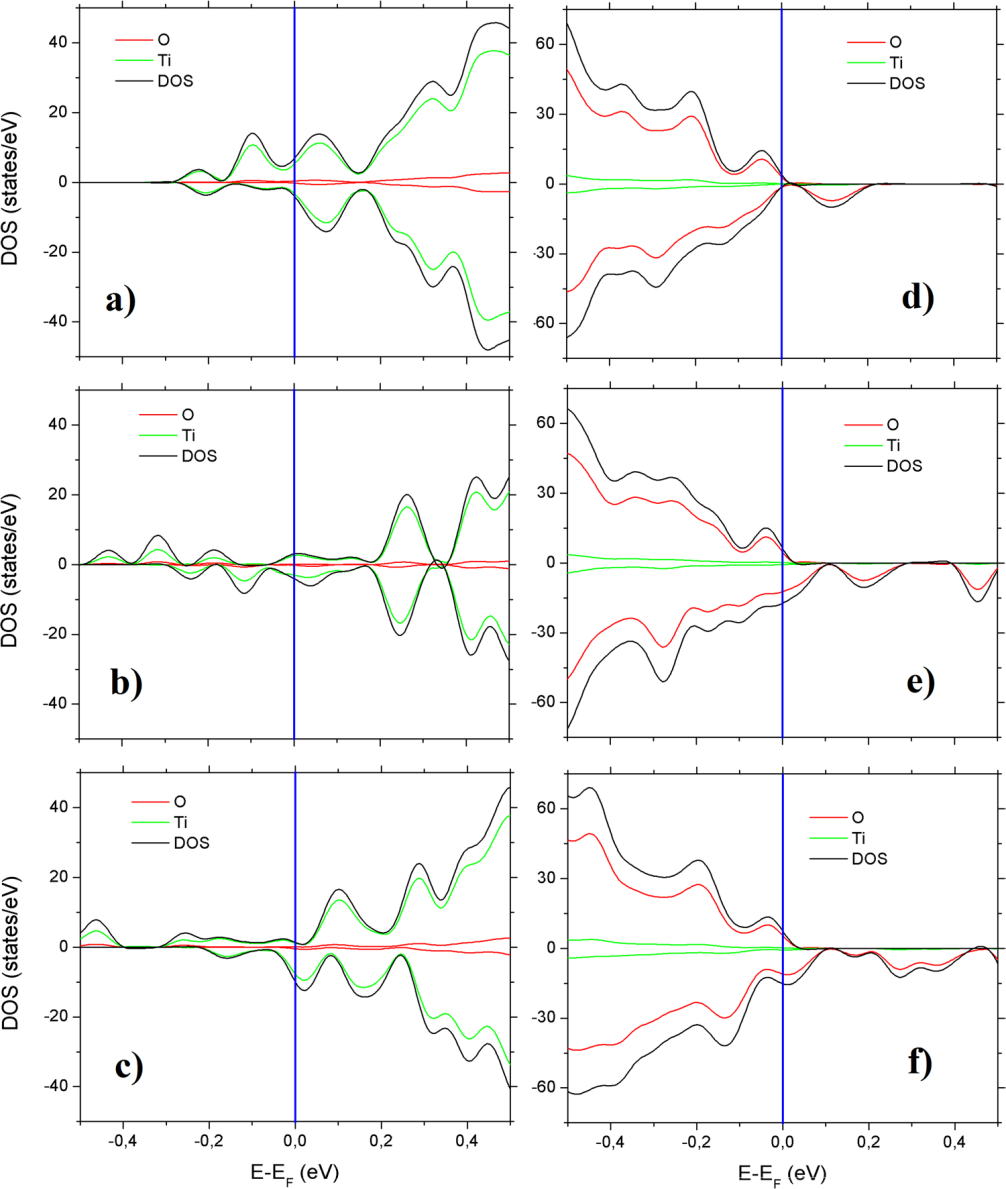
Density of states (DOS) in the Fermi zone for (**a**) 1-O, (**b**) 2-O, (**c**) 3-O, (**d**) a-Ti, (**e**) b-Ti, (**f**) c-Ti. In green and red are represented the titanium and oxygen contributions, respectively.

A scheme of the synthesis mechanism from the titanium *n*-propoxide precursor to the TiO_2_-QDs via water vapor flow diffusion is depicted in Fig. 1a. X-ray diffraction pattern (XRD) for TiO_2_-QDs is shown in Fig. 1b. The broad peaks at 2θ = 25.4, 38.1, 47.9, 54.6, 62.8, 69.4 and 75.3°, are assigned as (101), (103)(004)(112), (200), (105)(211), (213)(204), (116)(220) and (215)(301) planes, respectively^12^. The mean diameter crystallite size, estimated applying the Scherrer equation D = 0.9λ/βcos(θ) using the 2θ = 25.4° peak of the XRD pattern, was D = 5.0 nm^13^. Raman spectrum of the TiO_2_-QDs is shown in Fig. 1c. The typical TiO_2_ anatase Raman peaks are situated at ω ~ 151, 401, 518 and 636 cm^−1^, ascribed to E_g_, B_1g_, A_1g_ and E_g_ modes, respectively. The position and width of these Raman peaks is in good agreement for those found for TiO_2_ anatase with D ~ 5–6 nm mean crystallite size, due to phonon confinement effects^14–16^. It has been already observed that the presence of defects leads to extra symmetric broadening, particularly for the ω ~ 636 cm^−1^ mode, for which a total broadening as high as Δω ~ 60 cm^−1^ was detected^15^. In our case, we observe a total broadening of Δω ~ 80 cm^−1^ for the peak at ω ~ 636 cm^−1^, suggesting the presence of defects in our TiO_2_ quantum dots, which showed a similar crystallite size as those from literature^15^. We also distinguish two sharp peaks located at ω ~ 486 and 1467 cm^−1^, that could be assigned with the Ti-OPr stretching mode and CH_2_/CH_3_ bending mode, respectively. The notorious blue shift and sharpening of the Ti–OR stretching mode from ~ 430 to 480 cm^−1^ and the CH_2_/CH_3_ bending mode from ~1442 to 1464 cm^−1^, revealed that these modes are not related to TTP precursor, as shown in Fig. 1c. The presence of these well-defined Raman peaks could be suggesting the presence of residual *n*-propoxide groups, possibly bonded to the surface of the TiO_2_-QDs, as we showed in the scheme presented in Fig. 1a. In this scenario, the presence of this bonded *n*-propanoxide moiety is notorius at long times of synthesis regardless the effect of the lack of time of water vapor exposure. The functionalization of the surface itself could be also stopping the growth of TiO_2_-QDs, as similarly observed in the presence of polymers hosts^17–20^. High resolution transmission electron microscopy (HR-TEM) image is shown in Fig. 2a. The periodic atoms array observed in the HR-TEM image could be in relation to the (101) plane, whose associated estimated interplanar spacing is 0.352 nm, in good correlation with XRD analysis. The TiO_2_ quantum dot shown in Fig. 2a showed a quasi-spherical shape with a diameter estimation of ~5–6 nm, in strong agreement with the more representive mean particle sizes estimated using X-ray diffraction and Raman spectroscopy analyses, as discussed above. Small angle X-ray scattering (SAXS) curves collected for TiO_2_-QDs powdered samples are shown in Fig. 2b. In addition, a dilution of 10% in weight of TiO_2_-QDs in a polymer host was also collected as the reference of a diluted and poor-interacting TiO_2_ quantum dots system. The total SAXS intensity I(q) for highly concentrated scatterers obeys the following equation:

1 $$I(q)=\phi P(q)S(q)$$

where *φ* is the volume fraction, *P(q)* is the form factor ascribed to the particles and *S(q)* is the structure factor related to the interference function. Theoretically, the structure factor expression is described by the following equation:

2 $$S(q)=1+4\pi n{\int }_{0}^{\infty }[g(r)-1]{r}^{2}\frac{\sin (qr)}{qr}dr$$

where *g(r)* is the pair correlation function. The structure factor S(q) was obtained experimentally using the following equation: S(q) ~ I(q)/I_10%_(q), in which I(q) is the intensity of TiO_2_-QDs concentrated sample and I_10%_(q) is the intensity of the diluted system, assuming that I_10%_(q) is only attributed to the scattering of non interacting TiO_2_ quantum dots. S(q) vs. q curves fitted well using a perturbative solution of the Percus-Yevick closure, according to the sticky hard sphere (SHS) approximation proposed by Baxter^21^. We have previously reported the presence of coulombic repulsive interactions in the diluted regime of TiO_2_ quantum dots embedded in a polymer host prepared by the same methodology. However, our SHS approximation is suggesting the presence of short-range attractive interactions, that are typically observed in the concentrated regime^22^. Although we should not neglect that other non-magnetic short-range attractive interactions could be present, such as attractive capillary forces between TiO_2_ quantum dots^22^, the origin of these attractive interactions could be also related to the presence of dipole-dipole interactions, as already observed for other magnetic nanoparticles systems^23–27^. The magnetization versus applied magnetic field curves are presented in Fig. 3a. The magnetization saturation is observed at low applied magnetic fields (H ~ 2 kOe) at low temperatures (T = 5 K) and almost the same behavior is observed at room temperature (T = 300 K). Our results agree with a previous report of ferromagnetic titanium-defected undoped anatase TiO_2_ samples, but our saturation magnetization is about 0.0025 emu/g, which is approximately one order less than the reported in literature^6^. The presence of a small hysteresis loop and a coercive field is also evidenced, suggesting the presence of possible ferromagnetic interactions at low temperatures which are maintained at room temperature. Such kind of weak ferromagnetism behavior has been reported for undoped transition metal oxides and usually attributed to non-uniform magnetization and non-percolated large magnetic moments^28,29^. The presence of ferromagnetic interactions could be associated to the presence of uncompensated spin moments in titanium or oxygen vacancies possibly located at the interphase but also at the bulk of the TiO_2_-QDs^4,6^. However, due to the low dimensions of our TiO_2_-QDs point defect, mediated magnetic coupling leading to large magnetic moment particle cannot be discarded. The impedance response of our TiO_2_-QDs was obtained by applying an A.C. voltage with 300 mV amplitude added to a D.C. voltage of 3 V. The use of V_DC_ < 3 V applied DC biases showed a poor signal-to-noise ratio in our impedance measurements, basically due to the TiO2 anatase semiconductor electronic band gap in addition to the presence of large amounts of grain boundaries. Moreover, the use of similar DC voltages in addition to AC voltages in impedance studies were also performed for other semiconductor materials to study magnetic field effects on the impedance response^30,31^. The external D.C. magnetic field was applied externally using an electromagnet in the same direction of the applied voltage and ranged from 0 to 1800 Oe. The total impedance can be represented as:

3 $$Z=Z^{\prime} -iZ^{\prime\prime} $$

with Z′ being the real part and Z″ the imaginary part of complex impedance. The dependence of Z′ and Z″ with frequency for selected applied magnetic fields is shown in Fig. 3b. At first inspection, both real and imaginary components of impedance major changes were evidenced at low frequencies (f < 2000 Hz) with increasing applied magnetic fields, as already observed for other semiconductors^30,31^. However, in our case, other smaller changes for the impedance response are also observed with the applied magnetic field at intermediate frequencies. In Fig. 3c, we show both real and imaginary impedance as function of the applied magnetic field for different selected frequencies. It is clearly observed that the major changes are observed at 100 Hz, while almost no variations were observed at intermediate frequencies; i.e. 1 kHz and above. For this reason, we gave special attention to the circuit model which best describe the electrical transport of our TiO_2_-QDs samples. Impedance spectroscopy data and fitting curves for different applied magnetic fields, represented as Nyquist plots (−Z″ versus Z′), are shown in Fig. 4. Impedance data showed best fitting by using the circuit model determined by the sum of two contributions, each composed by parallel resistance and pseudo-capacitor usually called as constant phase element (CPE), connected in series, as shown in the left panel of Fig. 4. The impedance of the CPE element can be described using the following equation:

4 $$\frac{1}{{Z}_{CPE}}=Q{(i\omega )}^{n}$$

where *Q* is the numerical value of admittance at *ω* = 1 rad.s^−1^, *ω* is the frequency and *n* is an exponential factor. In the case of *n* = 1, the CPE resembles a capacitor and *Q* = C. These two contributions are related to two notorious arcs in the Nyquist plots; the smaller ones, observed at higher frequencies, possibly associated with the intrinsic electrical transport at the bulk of TiO_2_-QDs and the largest, observed at lower frequencies, possibly related to the electrical transport through the grain boundaries of TiO_2_-QDs, as already observed for TiO_2_ films in a systematic study^32^. In the Nyquist plots, the maximum of the arc associated frequency is related to relaxation frequency of the charge transfer processes. In our case, the associated relaxation frequencies of the smaller arc observed at higher frequencies showed no variations from ~45 kHz with increasing the applied magnetic fields, while the one corresponding to the larger arc at lower frequencies were at ~150–100 Hz, as depicted in Fig. 4. Impedance variations with applied magnetic field mostly observed at low frequencies and absence of variations in the high frequency regime discards mismatch of impedance originated by unwanted resonance effects. Moreover, while the arc ascribed with the grain contribution (higher frequencies) remains almost invariant with the applied magnetic field, the one related with the grain boundary contribution (lower frequencies) changes notoriously by increasing its size when the magnetic field increases. Both contributions of grain (R_g_) and grain boundary (R_gb_) resistance values obtained from the fitting and corresponding magnetoresistance [MR = 100(R_H_−R_0_)/R_0_] as a function of the applied magnetic field are shown in Fig. 5a. The R_g_ values showed only small variations in the whole range of applied magnetic fields, while R_gb_ showed a significant increment with increasing applied magnetic field yielding positive MR values up to +1200% at H = 1.8 kOe. The origin of the grain positive magnetoresistance in these diluted magnetic semiconductors can be explained based in Zeeman splitting increment in the presence of an applied magnetic field, which leads to the suppression of the hole’s hopping path^10,33^. The spin-splitting of the conduction band can be a consequence of the p-p exchange induced from the coupling between hole carriers and the magnetic moment induced by the polarized oxygen atoms in the surrounding of a Ti vacancy, as already reported for other undoped oxide semiconductors^10,33^. However, the major contribution to the magnetoresistance was attributed to the grain boundaries resistance, whose large increment with increasing applied magnetic fields is a well-known characteristic of the activated transport regime at high temperatures^34^. In addition, Coey *et al*. has also explained the presence of a non-typical ferromagnetic ordering on the regions containing defects such as grain boundaries in non-magnetic oxides in terms of the charge transfer magnetism^28^. On the other hand, both contributions of grain (Q/CPE_g_) and grain boundary (Q/CPE_gb_) pseudo-capacitance fitted values and corresponding magnetocapacitance [MQ = 100(Q_H_−Q_0_)/Q_0_] as a function of the applied magnetic field are shown in Fig. 5b. In this case, the Q/CPE_g_ contribution did not show drastic variations with the applied magnetic field, in analogy with the grain resistance behavior. However, the Q/CPE_gb_ contribution showed a decrease with increasing the applied magnetic field yielding negative MQ values up to −115% at H = 1.8 kOe. The presence of simultaneous large positive magnetoresistance and negative magnetocapacitance response was already observed for ferromagnetic tunnel junctions by using insulating MgO thin layer barriers^35^. However, up to now, there has been no reports on the granular magnetoresistance and magnetocapacitance response of undoped TiO_2_ quantum dots. In order to shed some light on the possible mechanism, we studied the magnetism and electronic properties of TiO_2_ (101) surface and subsurface oxygen and titanium vacancies by means of GGA + U calculations. The scheme of the titanium and oxygen vacancies is shown in the Fig. S1, where 1, 2 and 3 represents the surface, subsurface and third neighbor vacancies, respectively. All vacancies produced distortion in the TiO_2_ structure generating different statistical distribution in titanium-oxygen bond lengths, see Fig. S2. The formation energy was determined according to: E_form_ = E_vac_ − (E_TiO2_ + E_a_), being E_vac_ the total energy of the system with the vacancy, E_TiO2_ the energy of the pure ultrathin sheet of TiO_2_ and E_a_ the total energy of the isolated oxygen or titanium atom when corresponds. The formation energy and magnetic moment for all structures are summarized in Table 1. The lowest formation energies are observed for surface vacancies for both, oxygen and titanium. According to this, the removal of the corresponding atoms was easier, in relative terms of energy, than the subsurface and third neighbor vacancies. Moreover, if we compare surface vacancies of oxygen and titanium, the formation energy is lower for oxygen than for titanium atoms vacancy. This agrees with the observed trend in the titanium-oxygen bond length distribution: when the vacancy is generated by the oxygen extraction the distribution is less dispersed than the one generated by the titanium atom vacancy, as it can be seen in Fig. S2. While the magnetic moment for titanium vacancies are 4.00 µ_B_ without differentiating the vacancy position, the magnetic moment for oxygen vacancies are dependent on the atom vacancy position. Thus, in the case of the oxygen atom vacancy, the surface, subsurface and third neighbor cases exhibit a magnetic moment of 0.86, 0.02 and 0.98 µ_B_, respectively. Similar trend for the magnetic moment and formation energy are observed, for the subsurface vacancy the formation energy is the higher and the magnetic moment is the lowest one. The spin-density distributions for oxygen and titanium vacancy system, generated as the difference between spin-up and spin-down densities, are shown in Fig. 6. Although the magnetic moments for all titanium vacancy structures are the same, the spin-densities look very different, with spin-density concentrated near the surface for a-Ti and b-Ti, and more located in the middle of the ultrathin sheet for the c-Ti case. The partial charge density distribution shown in Fig. 7 help us to identify the origin of the electronic states generated close to the Fermi level when vacancies are created. In the case of O atom vacancies, there is an important contribution of Ti-d states in the region between −0.2 to 0.0 eV. In the case of Ti atom vacancies, there are relevant contributions from O-p states in the region between 0.0 to 0.20 eV. All these results are in concordance to the expected results, in which the valence band is mainly contributed by Ti-d states, while the conduction band is mainly contributed by O-p states. The density of electronic states (DOS) is presented in Fig. S3 and the corresponding zoom in the region close to the Fermi level in Fig. 8. There is an important asymmetry in the DOS as expected for evidenced spin unbalance when a vacancy is created, particularly higher in the case of Ti atom vacancies. One additional feature, is the metallic behavior in all vacancy structures, as it can be seen in Fig. 8. The atom removal, introduces impurity states close to the Fermi level, that goes into a further relaxation by creating a spin unbalance, thus generating a net magnetic moment. According to these results, the nature of the atom vacancy and the relative position in the ultrathin sheet contributes differently to the net magnetic moments, thus explaining some trends in the experimental data presented in this work.

**Table 1:** Formation energies and magnetic moments for all the structures.

Structure	E_FORM_ (eV)	Magnetic moment (µ_B_)
1-O	7.44	0.86
2-O	8.81	0.02
3-O	8.39	0.98
a-Ti	19.53	4.00
b-Ti	20.04	4.00
c-Ti	20.36	4.00

## Conclusions

In the present report, we showed a study on the large magnetoresistance and magnetocapacitance of TiO_2_ quantum dots prepared by sol-gel via water vapor diffusion method. Our structural characterization revealed the presence of TiO_2_ anatase quantum dots with a ~5 nm size with *n*-propoxide residuals possibly bonded to its surface. We also report a diluted ferromagnetic behavior probably due to the presence of vacancies, mainly at the grain boundaries of TiO_2_ quantum dots. The magnetoresistance and magnetocapacitance response associated to the grain boundaries, studied by means of impedance spectroscopy, showed MR = +1200% and MQ = −115%. Based in our study, we can conclude that the presence of defects, mainly at the grain boundary of these undoped TiO_2_ quantum dots, could be responsible of the large magnetoresistance and magnetocapacitance response at low applied magnetic fields.

## Methods

### Preparation of samples

Titanium oxide quantum dots (TiO_2_-QDs) were synthetized following the water vapor diffusion technique^17^. An amount of 0.5 g of titanium tetrapropoxide (TTP) 98% (purchased from Sigma-Aldrich) was suspended in 20 mL of tetrahydrofuran (THF) and 1 mL of deionizated water (H_2_O) and kept stirred until dryness at 60 °C. The resulting powder was ground and exposed to deionizated water vapor during 16 hours at 80 °C with a vapor flow of approximately 1 mL/min. Then the sample was dried at 80 °C for 7 hours in order to eliminate the residual propanol and water. Finally, the powder was ground and compressed at 50 kN/cm^2^ pressure to form pellets with a 1 cm diameter and 1.5 mm height.

### Characterization of samples

X-ray diffraction (XRD) was performed using a Rigaku Ultima IV diffractometer working in Bragg-Brentano configuration, with CuKα radiation, in the 2θ = 10–80° range with a step of 0.02° and 8 seconds integration time per step. The sample was studied by confocal Raman spectroscopy using WITec Alpha 300-RA with and excitation laser of 532 nm wavelength. Small angle X-ray scattering (SAXS) was performed at SAXS-1 Beamline Station as implemented at Laboratório Nacional de Luz Síncrotron in Campinas, Brazil (LNLS-CNPEM). The measurement was taken working in the q = 0.1–4.0 nm^−1^ range with 8 keV radiation at T = 300 K. The particle size and morphology were characterized using a high-resolution transmission electron microscopy (HR-TEM), JEOL 2100 instrument working under a 200 kV voltage. AC impedance spectroscopy analysis was performed using a Gamry Reference 3000 impedance analyzer at T = 300 K. The pressed pellet, with a 1 cm diameter and 1.5 mm height, was sandwiched between sputtered gold electrodes and impedance measurements were performed under a 3 V applied DC voltage and 300 mV AC voltage amplitude in the 1 Hz^−1^ MHz frequency regime. The applied DC magnetic field obtained from an electromagnet was applied parallel to the applied voltage and was varied in the H = 0–1800 Oe range. Magnetic measurements were taken using Quantum Design MPMS XL SQuID magnetometer by means of magnetization versus applied field (M vs. H) at 5 and 300 K with applied field in the H = 0–5 T range.

### Computational methods

We constructed ultrathin sheets of titanium dioxide, utilizing a TiO_2_ (101) surface, in which we generated subsurface and third neighbor oxygen and titanium vacancies, named as 1-O, 2-O, 3-O and a-Ti, b-Ti, c-Ti, respectively. We utilized a supercell with cell parameters a = 10.5 Å and b = 7.5 Å. In order to avoid interactions between adjacent images, we included a vacuum distance of 15 Å. First principles calculations were performed utilizing the VASP code (Vienna *Ab initio* Simulation Package)^36–40^, based on the density functional theory^41,42^ (DFT), utilizing the projector-augmented-wave (PAW) method^43^ and the generalized gradient approximation (GGA) as the exchange-correlation (XC) functional according to Perdew-Burke-Ernzerhof (PBE)^12,44^. The plane wave cutoff energy of 400 eV was employed and a regular Monkhorst-pack grid of 4 × 4 × 1 k-points was adopted in order to sample the full (reducible) Brillouin Zone. In all the cases a full structural optimization was performed. The unit cell parameters and atoms positions were adjusted until reaching the corresponding tolerance in the elements of the stress tensor and total forces down to 1 kBar and 0.01 eV/Å, respectively.

## Supplementary information

Supplementary information

## Data Availability

All data generated or analysed during this study are included in this published article (and its Supplementary Information files).
