# Supplementary material for: Extremely Large Magnetic-Field-Effects on the Impedance Response of TiO2 Quantum Dots
Source: Sci Rep. 2019 Mar 29;9:5322. doi: 10.1038/s41598-019-41792-z (PMC6440945; doi:10.1038/s41598-019-41792-z)
Supplement: Supplementary file 1 — Supplementary information [file 41598_2019_41792_MOESM1_ESM.docx]

**Supporting Information**

**Extremely Large Magnetic-Field-Effects on the Impedance Response of TiO_2_ Quantum Dots**

Dominique Mombrú^†^, Mariano Romero^†,^*, Ricardo Faccio^†,^*, Milton A. Tumelero^‡^ and Alvaro W. Mombrú^†,^*.

^†^Centro NanoMat/CryssMat & Física, DETEMA, Facultad de Química – Universidad de la República (UdelaR), Montevideo C.P. 11800, Uruguay.

^‡^Instituto de Física – Universidade Federal do Rio Grande do Sul (UFRGS), Porto Alegre C.P. 91501-970, Brazil.

(*) corresponding authors:

Prof. Dr. Mariano Romero (mromero@fq.edu.uy)

Prof. Dr. Ricardo Faccio (rfaccio@fq.edu.uy)

Prof. Dr. Alvaro W. Mombrú (amombru@fq.edu.uy)

The scheme of the titanium and oxygen vacancies is shown in the **Figure S1**, where 1, 2 and 3 represents the surface, subsurface and third neighbor vacancies, respectively. All vacancies produced distortion in the TiO_2_ structure that is why the titanium-oxygen bond length had a different distribution as shown in **Figure S2**.


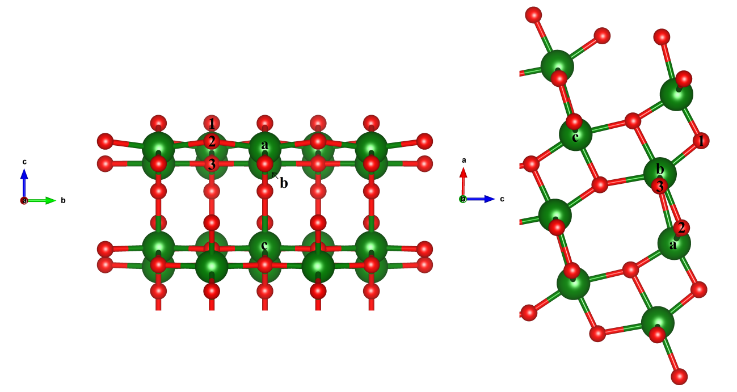


**Figure S1** – Scheme of the vacancies generated for oxygen (red) -1, 2 and 3- and titanium (green) –a, b and c-.

The density of states (DOS) graphics are shown in **Figure S3**. The spin-up and spin-down contributions of the density of states are different in all the graphics, especially for titanium vacancy system.


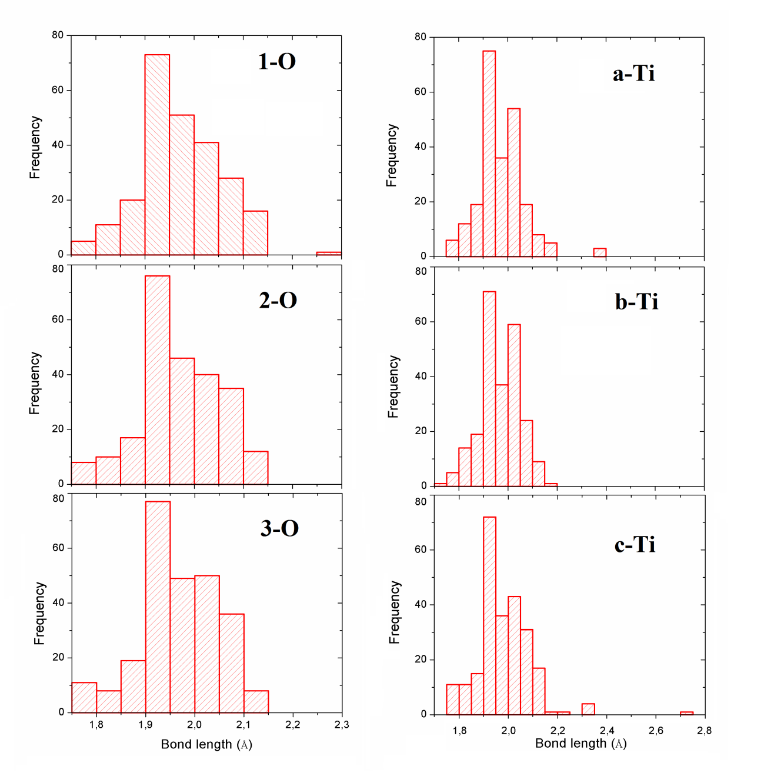


**Figure S2** – Histograms of titanium-oxygen bond length for all vacancy structures.


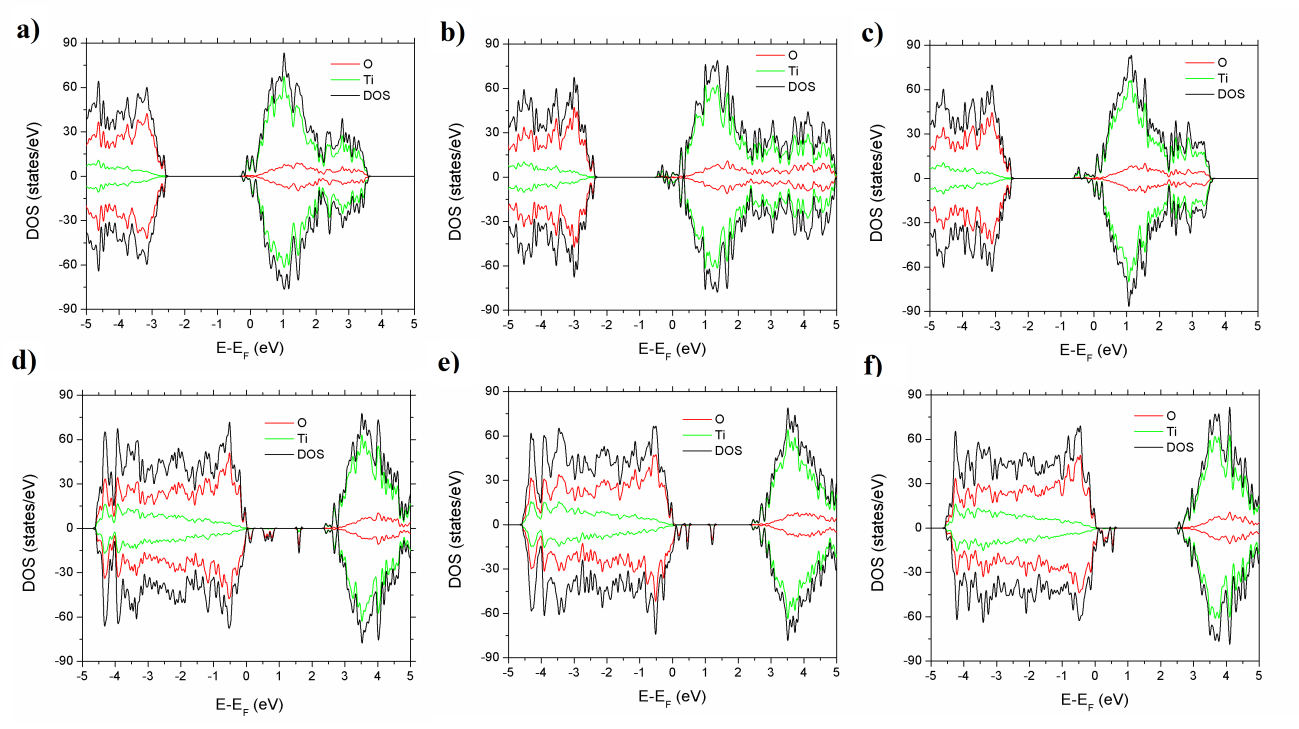


**Figure S3** – Density of states (DOS) for a) 1-O, b) 2-O, c) 3-O, d) a-Ti, e) b-Ti, f) c-Ti. In green and red are represented the titanium and oxygen contributions, respectively.
